# Supplementary figures and images for: Congenital syphilis in Argentina: Experience in a pediatric hospital
Source: PLoS Negl Trop Dis. 2021 Jan 6;15(1):e0009010. doi: 10.1371/journal.pntd.0009010 (PMC7815155; doi:10.1371/journal.pntd.0009010)

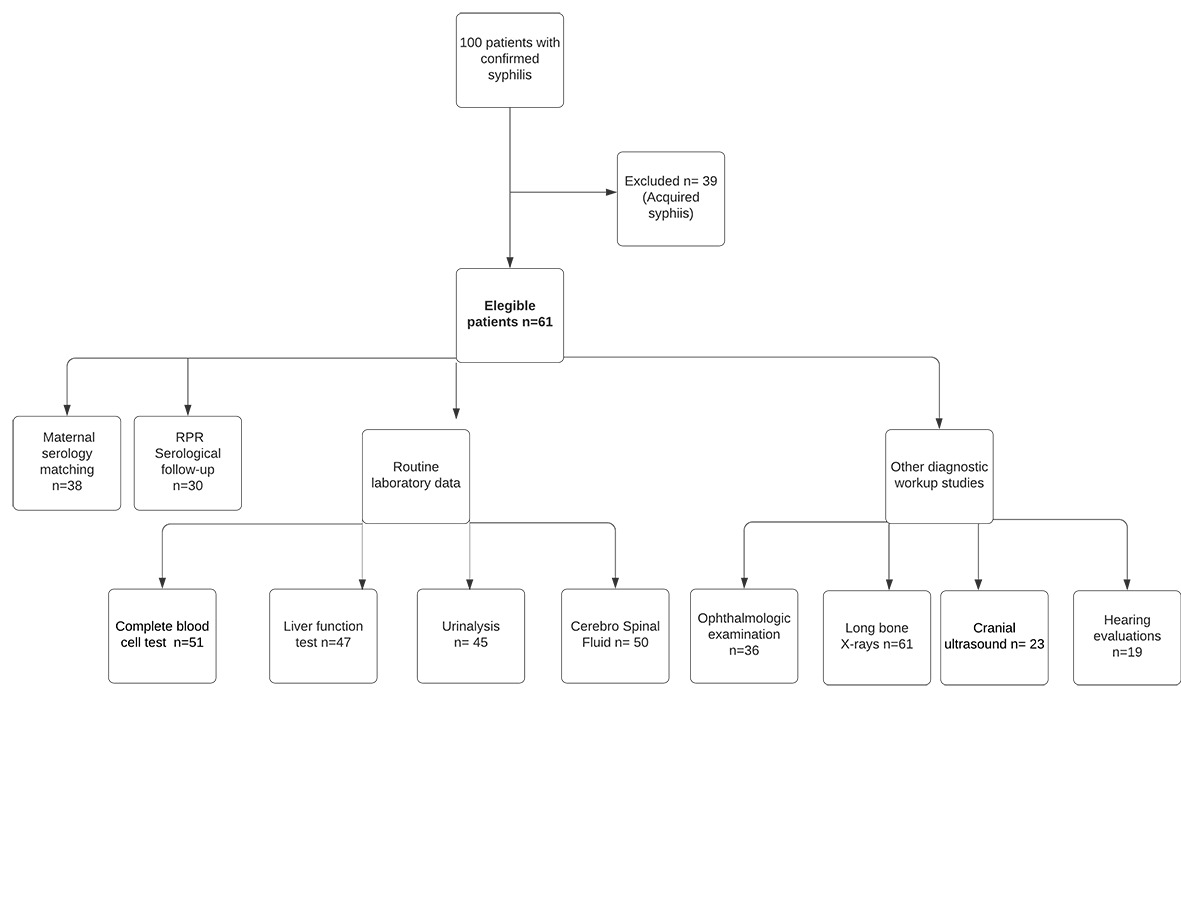

Supplement: S1 Fig — (TIF) [file pntd.0009010.s001.tif]

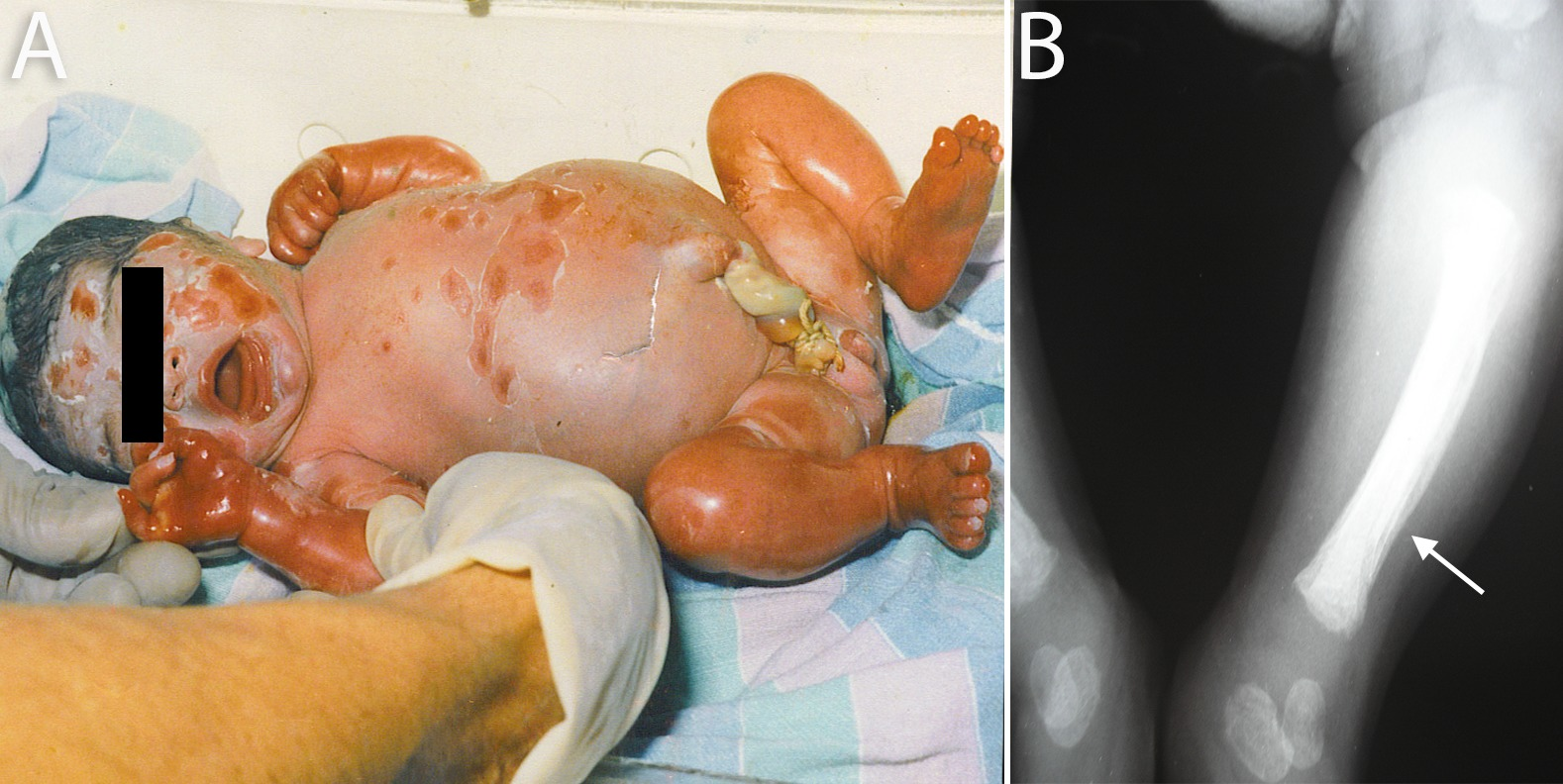

Supplement: S2 Fig — A) Term male newborn, adequate for gestational age with widespread erythroderma, vesiculobullous eruptions and desquamation (pemphigus syphiliticus). (B) Bone involvement lesions in the same patients are shown (arrow). (TIF) [file pntd.0009010.s002.tif]
