# Supplementary material for: Congenital syphilis in Argentina: Experience in a pediatric hospital
Source: PLoS Negl Trop Dis. 2021 Jan 6;15(1):e0009010. doi: 10.1371/journal.pntd.0009010 (PMC7815155; doi:10.1371/journal.pntd.0009010)
Supplement: Attachment — Submitted filename: Response to Reviewers.docx [file pntd.0009010.s004.docx]

**Reviewers' Comments to the Authors:**

**Reviewer 1**

**Summary and General Comments: Authors compiled a large case series of congenital cases in Argentina from 1987 through 2019. The clinical description, follow ups and treatments are very well described and of clinical interest for the medical community. Few minor revisions can improve the main message of the manuscript**

Author response: Thank you for pointing this out. We are grateful for the insightful comments on and valuable improvements to our paper

- **Abstract: Please adjust to mention the sequelae at follow ups. This is perhaps one of the most relevant findings of the study**

Author response: This was corrected as suggested. Lines 33-34.
- **Results: Please expand on the clinical findings of; "In 4 patients persistent proteinuria was observed as a sequelae of renal involvement and in 1 patient knee arthrosis occurred due to bone damage". Was renal failure observed? what age were those 4 patients? was the proteinuria attributed to congenital syphilis?**

Author response: We have added the suggested content to the manuscript on lines 220-233, and Fig 6
- **Discussion: Please expand on reasons why only 47% of pregnant women completed an adequate syphilis screening? What public health measures can be implemented to link those pregnant women to care? Is it routine to screen newborns and infants for Congenital syphilis born who were born without any prenatal care?**

Author response: The data was expanded, as suggested: Lines 293-300, 398-405 and 382-386, respectively.

- **Discussion: As a reader, I'd like to hear about more actionable recommendations in your community to prevent additional cases of congenital syphilis.**

Author response: We have added the suggested content to the manuscript on lines 406-418

**Reviewer 2**

- **Methods: The main objective of the study is to describe a Buenos Aires (Argentina) Children's Hospital congenital syphilis experience over a period of 32 years (1987-2019). To do so the study team conducted a retrospective medical record review of all patients diagnosed with presumed congenital syphilis over a 32 year time period .
  In this reviewer's opinion, this study lacks scientific rigor and attention to detail.  The study lacks clarity in the case definition of congenital syphilis.   This is a major flaw of this study and in this reviewer's opinion it makes the unsuitable for publication in its current format.**

Author response: We appreciate the reviewer’s feedback, in order to improve clarity we have detailed the case definition of congenital syphilis and contrasted to international and local guidelines. Lines 87-98

- **Results: This manuscript is poorly written.  It lacks coherence and it is truly impossible to read without getting confused and distracted. Tables and fixtures are of poor quality and denote a lack of attention to detail, which is not simply a lack of command of the English language.**

Author response: As the reviewer suggested, we have carefully reviewed and reinforced the writing throughout the manuscript. Widespread change made, for instance, switching the order in paragraphs and figures are highlighted in the revised manuscript file. Tables and figures adhere to the quality formatting rules and constraints requirements at submission, specified in https://journals.plos.org/plosntds/s/tables and <https://journals.plos.org/plosntds/s/figures>. In this way, captions are in the manuscript while figures are submitted as separated files. In order to improve clarity tables and some figures has been simplified and updated as well as captions.

As a comment, over the past 5 years, operational tools have been developed at our hospital to help digitally document data, while X-ray images are not yet computerized. Most of the images presented in the figures have the picture quality correspond to radiographies and image films, as we keep storages ours clinical charts.

- **Conclusions This paper is a hastily completed description of CS cases over a 30 year period.  The author's did not attempt at correlating their findings to historical data, epidemiological trends in Argentina or Latin America, or the many papers that have been published over the past decade describing congenital syphilis throughout the world.  Based on the manuscript it appears that the author's made no attempt at describing how they ascertained cases and if they believe their cases are a true estimate of all cases seen at their hospital.**

Author response: Although we agree that this is an important consideration, the lack of high-quality data about maternal and congenital syphilis in Latin America Countries has been concurrently reported by health organizations in compiled reports, making it difficult to correlate our retrospective findings with the local epidemiological trends. However, as suggested by the reviewer, we have contrasted and expanded with the available data: Lines 265-285 in Discussion.

The ascertained of our data and suggested contribution to strategies for control of the disease have been expressed in Discussion. Lines 406-418.

- **Editorial and Data Presentation Modifications: The methods section needs to be revised and strengthen and carefully described.  Tables and figures need to be revised and aligned with existing models for how to describe congenital syphilis.**

Author response: To improve clarity, the methods section has been expanded and revised, while tables and some figures, as well as figure captions, have been simplified and updated.

**Summary and General Comments:  In summary this study is not suitable for publication in its current format.**

Author response: We appreciate the opportunity to submit a revised draft of the manuscript. We have carefully considered and incorporated most of the suggestions made by the reviewer. We hope you find the new manuscript satisfactory and accurately.
